# Study on Fabrication of ZnO Waveguide Layer for Love Wave Humidity Sensor Based on Magnetron Sputtering

**DOI:** 10.3390/s18103384

**Published:** 2018-10-10

**Authors:** Changbao Wen, Taotao Niu, Yue Ma, Nan Gao, Feng Ru

**Affiliations:** Institute of Micro-Nanoelectronics, School of Electronics and Control Engineering, Chang’an University, Xi’an 710064, China; 2016132055@chd.edu.cn (T.N.); 2017232040@chd.edu.cn (Y.M.); 2017232036@chd.edu.cn (N.G.); fengru@chd.edu.cn (F.R.)

**Keywords:** Love wave device, humidity sensor, ZnO waveguide layer, magnetron sputtering

## Abstract

The ZnO waveguide layer for the Love wave humidity sensor was fabricated by radio frequency (RF) magnetron sputtering technique using ZnO as the target material. To investigate the effect of RF magnetron sputtering temperature on the ZnO waveguide layer and Love wave device, a series of Love wave devices with ZnO waveguide layer were fabricated at different sputtering temperatures. The crystal orientation and microstructure of ZnO waveguide was characterized and analyzed, and the response characteristics of the Love wave device were analyzed by network analyzer. Furthermore, a humidity measurement system is designed, and the performance of the Love wave humidity sensor was measured and analyzed. The research results illustrate that the performance of the ZnO waveguide layer is improved when the sputtering temperature changes from 25 °C to 150 °C. However, when the sputtering temperature increases from 150 °C to 200 °C, the performance of the ZnO waveguide layer is degraded. Compared with the other sputtering temperatures, the ZnO waveguide layer fabricated at 150 °C has the best c-axis orientation and the largest average grain size (53.36 nm). The Love wave device has the lowest insertion loss at 150 °C. In addition, when the temperature of the measurement chamber is 25 °C and the relative humidity is in the range of 10% to 80%, the fabricated Love wave humidity sensor with ZnO waveguide layer has good reproducibility and long-term stability. Moreover, the Love wave humidity sensor has high sensitivity of 6.43 kHz/RH and the largest hysteresis error of the sensor is 6%.

## 1. Introduction

Love wave, which is one of the shear horizontal waves, can be excited by the inter-digital transducer (IDT) deposited on a semi-infinite piezoelectric substrate and propagates in the waveguide layer [1,2]. In the Love wave device, the direction of the particle vibration of the Love wave is parallel to the surface of the substrate, and the energy is mainly concentrated in the waveguide layer. Because the coupling effect between the substrate surface and the waveguide layer load is very poor [3,4], the Love wave device can not only be used as a gas material sensor, but also can measure the humidity in the presence of liquid or gas-liquid coexistence, which other humidity sensors cannot do [5,6,7]. In addition, the Love wave humidity sensor has the advantages of fast response and small size. Therefore, it has been widely used in biosensor and analytical chip [8,9,10].

The Love wave humidity sensor requires that the shear velocities and densities of the waveguide layer materials are smaller than those of the piezoelectric substrate materials, and the waveguide layer materials were required to have good elastic properties and weak acoustic wave absorption performance [11,12,13]. At present, some materials can be used as a waveguide layer of the Love wave device, such as polyimide (PI), poly methyl methacrylate (PMMA), silicon dioxide (SiO_2_) and zinc oxide (ZnO) [14,15,16,17]. In these materials, ZnO is a kind of piezoelectric material with positive temperature coefficient, which can improve the electromechanical coupling performance, the conversion efficiency, and the temperature characteristics of the Love wave humidity sensor. Furthermore, the ZnO waveguide layer possesses advantages of high surface free energy, the strong adsorption capacity, the good biocompatibility and the good hydrophilic [18]. It can provide a good biological activity surface for biomolecules as a waveguide layer. Therefore, the Love wave device with ZnO waveguide have been applied to humidity sensors and biosensors [19,20].

The ZnO waveguide layer can be fabricated by various techniques, such as chemical vapor deposition [21], Sol-Gel process [22], and magnetron sputtering technique [23,24]. Although the chemical vapor deposition and Sol-Gel process have the advantages of the low cost and easy fabrication, the poor quality of film and the poor reproducibility are also presented. Thus, these two methods are not suitable for the fabrication of high quality and the large number of devices. Although the fabrication of ZnO waveguide layer by magnetron sputtering technique is complicated and expensive, the ZnO waveguide layer fabricated by this method has the advantages of high quality and good c-axis orientation [25]. Moreover, this method is a planar fabrication technique with low material cost and good adhesion ability of the substrate, so it can satisfy the requirements of large scale and industrial production of devices.

Based on these advantages of ZnO as the waveguide layer in the Love wave device and the characteristics of ZnO waveguide layer, a fabricated scheme of ZnO waveguide layer for the Love wave device based on magnetron sputtering technique is presented. The influence of sputtering temperature on ZnO waveguide layer and the Love wave device are analyzed by experiments. Furthermore, a humidity measurement system was designed to study some important characteristics of the Love wave device used as humidity sensor.

## 2. Schematic Diagram of Love Wave Humidity Sensor

**Figure 1:**
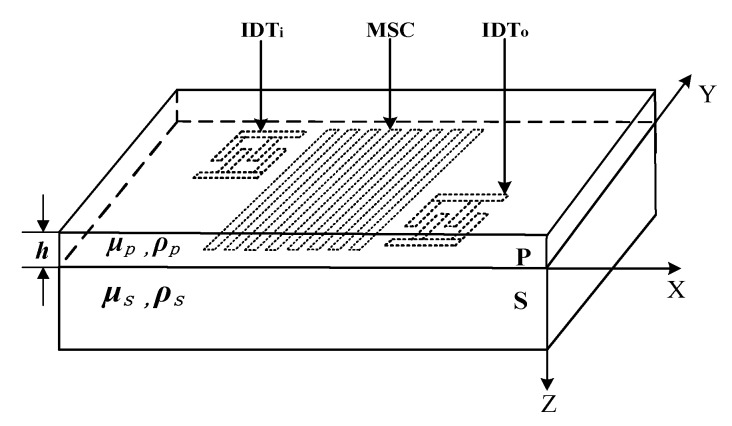
Schematic diagram of the Love wave humidity sensor.

Figure 1 shows the schematic diagram of the Love wave humidity sensor. Where S is a piezoelectric substrate and P is a ZnO waveguide layer. The IDT_i_ and IDT_o_ are the input and output transducer respectively, which are located on the piezoelectric substrate S and are covered in the ZnO waveguide layer P. The *μ_s_* and *ρ_s_* are the shear modulus and density of the piezoelectric substrate material, and the *μ_p_* and *ρ_p_* are the shear modulus and density of the ZnO waveguide material. The *h* is the thickness of the waveguide layer. The X, Y and Z are the three-dimensional coordinate parameters. The MSC is a multistrip coupler which consists of a set of parallel metal strips fabricated on the piezoelectric substrate. In the actual Love wave device, owing to inverse piezoelectric effects of piezoelectric crystals, the IDT_i_ excites not only the Love wave propagating along crystals surface, but also bulk acoustic wave (BAW) that has higher frequency than that of the Love wave signal. The BAW affects the performance of the sensor, such as producing some ripples and spurious components in response curve. According to the Reference [26], the Love wave excited by the IDT_i_ can be transferred to the lower half of the MSC under certain conditions while does not affect the propagation path of the BAW and output from the top half of the MSC. Therefore, we use MSC to separate the Love wave and BAW into two sound paths, which reduces the parasitic response of BAW and improves the performance of the Love wave device.

When the excitation signal is applied to the input transducer IDT_i_, a Love wave propagating in the ZnO waveguide layer P along the X-axis is generated by the inverse piezoelectric effect of the piezoelectric substrate S. Because the Love wave is a shear horizontal wave, when the semi-infinite substrate occupies the space of Z < 0 and the waveguide layer occupies the (0, *h*) area, the change in particle displacement only exists in the area of the (0, *h*), without any displacement components in the normal direction of the substrate. In this case, the energy loss is low.

When the Love wave humidity sensor is operating, the propagation velocity of the Love wave is affected by the mass of the material loading on the device, the applied electrical signal, the mechanical properties of the substrate material and the external environment factors. The intrinsic relationship as shown in following equation [27]:

(1) $$\frac{\Delta v}{v}=\frac{1}{v}\left[\frac{\partial v}{\partial_{mass}}\Delta_{mass}+\frac{\partial v}{\partial_{elec}}\Delta_{elec}+\frac{\partial v}{\partial_{stru}}\Delta_{stru}+\frac{\partial v}{\partial_{env}}\Delta_{env}\right]\;$$

where v is the propagation velocity of the acoustic wave in the device and Δv is the variation of acoustic propagation velocity. $\Delta_{mass}$ is the mass change of substance loading on the acoustic wave propagation path. $\Delta_{elec}$ is the variation of electrical signal applied to the device. $\Delta_{stru}$ is the variation of structural parameters of piezoelectric materials which include resistance and dielectric constants and so on. $\Delta_{env}$ is the variation of the environmental parameters of the device. In the Love wave device, the relationship between the acoustic wave propagation velocity and the center frequency of device is given by Equation (2):

(2) $$\frac{\Delta f}{f_{0}}=\frac{\Delta v}{v}\;$$

where ***f***_0_ is the center frequency of device, and Δ*f* is the frequency shift of the center frequency.

According to Equations (1) and (2), when the ZnO waveguide layer in the Love wave humidity sensor has an adsorption effect on the water vapor, the mass and structural parameters of the ZnO waveguide layer will change due to adsorbing the measured materials. The change will cause the change of acoustic wave propagation velocity in the Love wave device, and further leads to the change of the center frequency of the Love wave device. While the frequency shift is determined by humidity, thus the change in humidity can be measured indirectly by measuring the frequency shift.

## 3. Experiments

### 3.1. Fabrication of ZnO Waveguide Layer

The most commonly used piezoelectric crystal materials in Love wave devices are quartz, LiNbO_3_ and LiTaO_3_. Compared with quartz and LiTaO_3_, LiNbO_3_ has higher piezoelectric coefficient, electromechanical coupling coefficient and lower sound attenuation. Moreover, it is reported that the parasitic level of the Love wave is the lowest when the substrate is 128° YX-LiNbO_3_, so the Love wave device based on 128° YX-LiNbO_3_ has the lower insertion loss and better performance. Therefore, the 128° YX-LiNbO_3_ crystal with k^2^ = 5.5% is used as the piezoelectric substrate material of the device.

To suppress the effect of secondary load caused by the electrode quality, the aluminum (Al) with low mass and resistivity is chosen as the IDT electrode material. The Al films were fabricated by direct current sputtering technique and a pair of IDTs was fabricated on the 128° YX-LiNbO_3_ by the ultraviolet (UV) exposure method. The design parameters of the Love device are shown in Table 1.

The thickness of the ZnO waveguide layer affects the sensitivity and the insertion loss of the Love wave device [28,29]. To obtain higher sensitivity and lower insertion loss, the thickness of the device is designed to be 3 μm.

The ZnO waveguide layer was fabricated by the JGP450 magnetron sputtering system. The JGP450 magnetron sputtering system is manufactured by Shenyang scientific instrument Co., Ltd. Chinese Academy of Sciences, and it consists of a sputtering vacuum chamber, magnetron sputtering target, substrate water cooling heating table, working gas path, pumping system, installation machine, vacuum measurement, film thickness monitoring system, and electronic control system. Its limiting vacuum degree is not greater than 6.67 × 10^−5^ Pa. The target material is the ZnO with a purity of 99.99%. The sputtering parameters are listed in Table 2.

**Figure 2:**
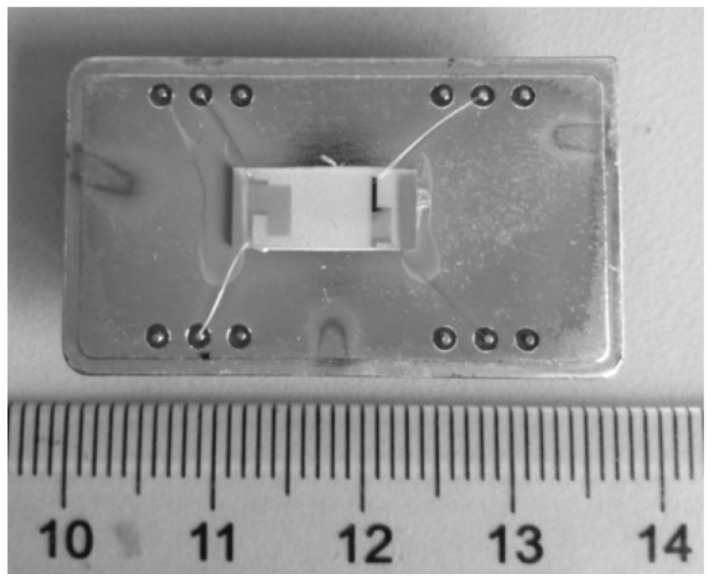
Love wave device with ZnO waveguide layer fabricated at 150 °C.

The fabrication process of the ZnO waveguide layer mainly includes four steps. Firstly, place the substrate and target. Secondly, pumping the vacuum of the sputtering chamber to 4.0 × 10^−4^ Pa by rough pumping and fine pumping. Thirdly, sputtering of the ZnO waveguide layer. The sputtering pressure is 1.2 Pa, the sputtering power is 120 W, and the sputtering time is about 200 min. Finally, complete the sputtering of the ZnO waveguide layer and take out the device. Figure 2 shows the Love wave device with ZnO waveguide layer fabricated at the sputtering temperature of 150 °C.

The ZnO waveguide layer fabricated by magnetron sputtering technique can be regarded as the process of gas atoms or atomic groups from the sputtering target agglomerate into solid on the substrate surface. Whether the growth process of the ZnO waveguide layer can be sustained or not, the nucleation rate is a key problem.

According to the Boltzmann equation, the nucleation rate of the ZnO waveguide layer can be written as:

(3) $$v_{n}=Zn_{1}2\pi r\sin\alpha ja_{0}\exp[(E_{d}-E_{D}-\Delta G)/k_{0}T_{s}]$$

where *Z* is the Zeldovich correction factor and its value is about 10^−2^. $n_{1}$ is the number of atoms adsorbed by the per unit area and *r* is the critical nuclear radius. *α* is the contact angle between the atomic groups and the substrate and $a_{0}$ is the distance between the adsorption sites. *j* is the intensity of the vaporized gas atoms which incident on the surface of the substrate. *E_d_*, *E_D_* and Δ*G* are the adsorption energy, surface diffusion energy and highest free energy, respectively. *k*_0_ is the Boltzmann constant and *T_s_* is the sputtering temperature.

From Equation (5), in the process of the ZnO waveguide layer fabricated by magnetron sputtering method, the sputtering temperature has an important effect on the nucleation rate of the ZnO waveguide layer and it determines the crystal orientation and crystal size of the ZnO waveguide layer. The nucleation rate will fast if the sputtering temperature too high. In this case, the atoms would not accomplish orderly lattice arrangement, which will affect the crystalline quality of the waveguide layer and the performance of the Love wave device. Therefore, how to control or select an appropriate sputtering temperature is the key to fabricating the high-performance Love wave device.

To research the effect of RF magnetron sputtering temperature on the crystal orientation, microstructure and stress of the ZnO waveguide and the influence in the performance of the Love wave device. A series of Love wave devices with the ZnO waveguide layer were fabricated under the sputtering temperatures of 25 °C, 50 °C, 100 °C, 150 °C and 200 °C, respectively. The others sputtering parameters as shown in Table 2. Then the crystal orientation and microstructure of the ZnO waveguide layer were analyzed by the X-ray diffraction instrument. The response characteristics of the Love wave device with ZnO waveguide layer were analyzed by network analyzer.

### 3.2. Design of the Humidity Measurement System

**Figure 3:**
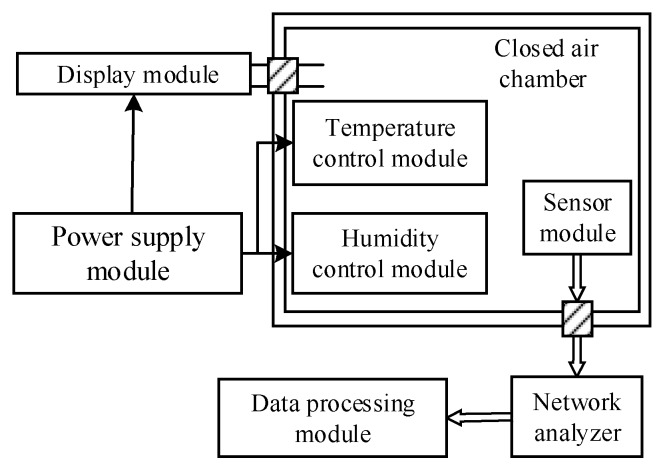
Structure diagram of humidity measurement system.

Figure 3 shows the structure diagram of the humidity measurement system. It mainly consists of the closed air chamber, the sensor module, the temperature control module, the humidity control module, the network analyzer, the data processing module, the power module and the display module.

**Figure 4:**
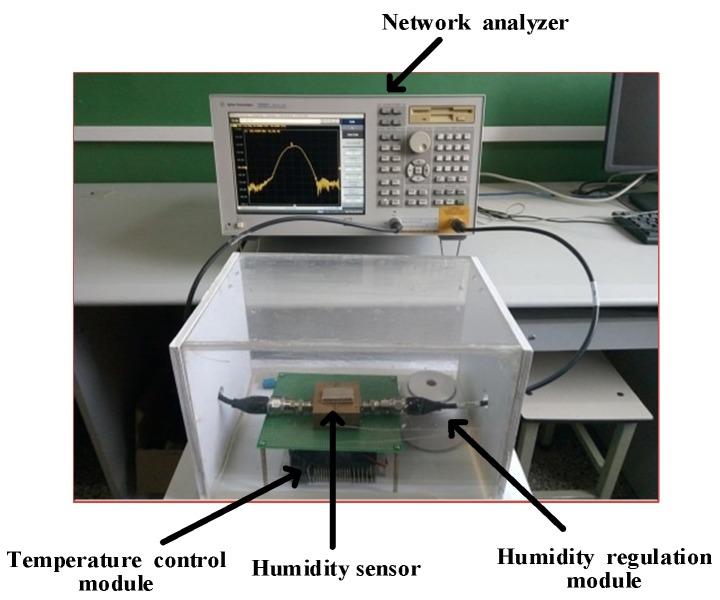
Part of the real measurement system.

As observed from Figure 3, the sensor module, the temperature control module and the humidity control module are in the closed air chamber. The sensor module is a Love wave humidity sensor with the ZnO waveguide layer fabricated at 150 °C. A 50-ohm matching is required at both ports of the device for proper connection which has low-reflection loss. The temperature control module is a semiconductor refrigerator and its control circuits. The humidity control module includes a humidification unit and a dehumidifying unit. The other components of the humidity measurement system are located outside the closed air chamber. In these modules, the power module mainly provides power for the display module, the temperature control module and the humidity control module. The display module is composed of the microprocessor and the DHT11 sensor which can measure the temperature and humidity simultaneously. The DHT11 sensor is a digital temperature and humidity sensor, and it is manufactured by waveshare electronics Co., Ltd. Shenzhen, China. When measuring temperature, the resolution is 1 °C, the accuracy is ±2 °C, and the detection range is 0 °C ~ 50 °C. When measuring humidity, the resolution is 1%RH, the accuracy is ±5 %RH, and the detection range is 10 %RH ~ 90 %RH. So we can simultaneously measure the temperature and humidity in the closed air chamber by the display module. The spectral characteristics of the sensor were measured by the Agilent E5062A ENA-L RF network analyzer which made in Malaysia and its measurement range is from 300 kHz to 3 GHz. The data processing module mainly accomplishes the storage and process of the relevant data. Part of the real measurement system is shown in Figure 4.

## 4. Experimental Results and Analysis

### 4.1. Crystal Orientation Analysis of the ZnO Waveguide Layer

**Figure 5:**
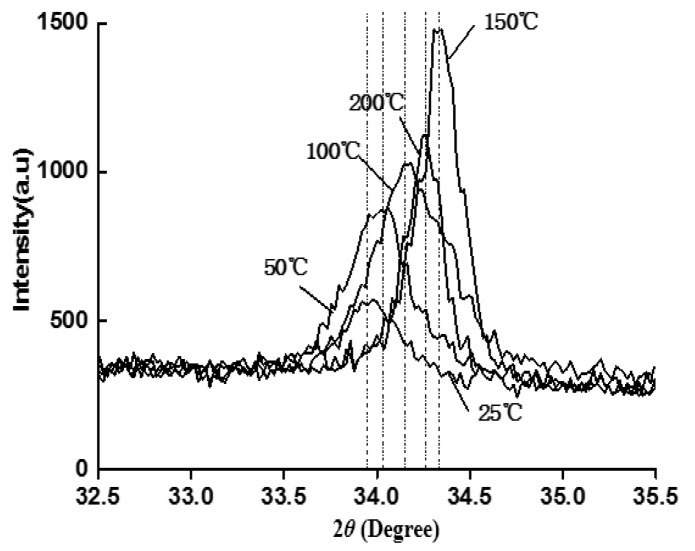
XRD spectra of the ZnO waveguide layer.

The c-axis orientation of ZnO waveguide layer has an important effect in the performance of the Love wave device. The magnetron sputtering technique can be used to fabricate the ZnO films with good c-axis orientation, high surface evenness and few defects. However, the crystal orientation of the ZnO waveguide layer would be different because of the different process parameters, such as the sputtering temperature and power. The X-ray diffraction instrument is used to analyze the ZnO waveguide layers which are fabricated at five different sputtering temperatures. The X-ray diffraction (XRD) spectrum of the ZnO waveguide layer is shown in Figure 5.

It can be seen from Figure 5 that the ZnO waveguide layers have the highest diffraction peaks near 34.4° at the five sputtering temperatures, and the peaks correspond to the non-stress powder (002) plane diffraction peak of ZnO. The results reveal that the ZnO waveguides growing on the LiNbO_3_ substrate have (002) preferred orientation at the five sputtering temperatures. That is, the growth of the c-axis is perpendicular to the surface of the substrate.

Furthermore, in the range from 25 °C to 150 °C, the (002) diffraction angles of the ZnO waveguide layers gradually approach 34.4° and the value of the peaks also gradually increase with the temperature rising. When the sputtering temperature goes up to 200 °C, the (002) diffraction angles are deviate from 34.4° and the peaks value are decreasing. This is because the critical nucleus radius of the film is very small and new nuclei will continue to be produced during the deposition process at a lower sputtering temperature. Moreover, the surface diffusion energy of the atoms or the groups adsorbed on the LiNbO_3_ is very low, and they cannot migrate to the positions of the lattice where energy are lowest, which leads to the structure of the waveguide layer become loose and emerge some holes of the nanometer scale. This waveguide layer has many disadvantages including many defects, poor c-axis orientation and weak relative intensity of the diffraction peak. These disadvantages eventually lead to the poor acoustic performance. With the increasing of the sputtering temperature, the diffusion of the adsorbed atoms is gradually become the main factor which is affecting the structure and morphology of the films. Atomic diffusion will reduce the number of holes caused by some factors, which will transform the film structure into a good columnar shape and the c-axis becomes better. When the sputtering temperature is too high, the adsorption lifetime of external particles is shortening, and the decomposition rate is increasing, which leads to the reduce of the crystal quality and the c-axis orientation, and eventually the relative intensity of the diffraction peaks become weaker.

### 4.2. Microstructure Analysis of the ZnO Waveguide Layer

From the theory of nucleation thermodynamics under the condition of non-spontaneous nucleation, the grain size is closely related to the sputtering temperature. To quantitatively analyze the crystallinity of the waveguide layer, the grain size *D* is estimated based on the Scherrer equation [30]. That is:

(4) $$D=\frac{K\lambda }{\eta \cos\theta }$$

where *K* = 0.94 is the Scherrer constant and *λ* = 0.1540598 nm is the wavelength of X-ray. *θ* is the diffraction angle and *η* is the full-width at half maximum of the diffraction peak. According to the full-width at half maximum of the diffraction peaks in the XRD spectra, the relationship between the different sputtering temperatures and the grain size can be calculated. The results are shown in Table 3. The sputtering time of the five experiments in Table 3 is 400 min. In the process of calculating grain size, because the broadening caused by the lattice distortion and instruments cannot be separated effectively, the calculated grain size may be smaller than the real size. 

As depicted in Table 3, when the sputtering temperature increases from 25 °C to 150 °C, the grain size also increases and reaches the maximum at 150 °C. If the sputtering temperature continuously increases to 200 °C, the grain size would reduce. In the growth process of the ZnO waveguide layer, it is difficult to form the crystal with large scale and regular morphology due to the influence of various factors. Thus, the waveguide layer is formed by the gather together of the ZnO particles. The energy of the internal crystal boundary and the performance of the film depend on the grain size. If the grain size is larger, the crystal boundary will be less, and the defects are fewer. In this case, the film quality is better. The ZnO waveguide layer is fabricated at 150 °C has the largest grain size, which indicates that the fabricated ZnO waveguide layer at 150 °C has highest crystallinity and best film quality.

**Figure 6:**
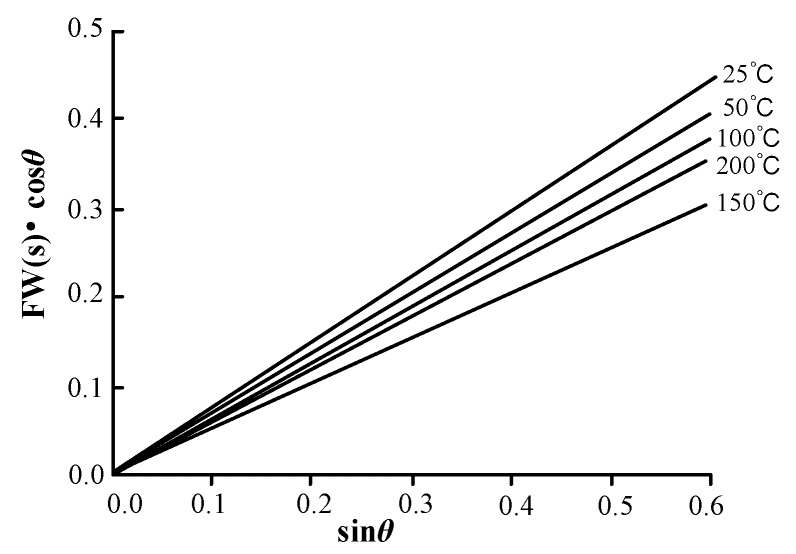
Microscopic strain graphic of the ZnO waveguide layer.

During the growth process of the ZnO waveguide, the stress may change the growth mode of the ZnO, which would affect the surface morphology and c-axis orientation of the ZnO waveguide layer. The stress would cause the micro-strain, which lead to an increase of the full-width at half maximum. We can assume that the increasing full-width at half maximum is FW(s) caused by micro-strain. To investigate the micro-strain of the ZnO waveguide layer fabricated at the different sputtering temperatures, the micro-strain curves at each sputtering temperature were analyzed. The result is shown in Figure 6.

As can be seen from Figure 6, although the five curves all pass through the origin, the slopes are different because the microscopic strain is different. At a lower sputtering temperature, the c-axis orientation of grains is not completely parallel caused by many holes in the ZnO waveguide layer. The growth of the columnar crystal is not strictly perpendicular to the substrate and the grains are squeezing with each other, which leads to the increase of the micro-strain. With the sputtering temperature increasing, the c-axis orientation of grains becomes better. Meanwhile, the stress along the c-axis direction and the micros-strain would reduce. These results show that the internal defects in the films are greatly reducing. The reason is that the adsorbent atoms have high diffusion energy and the atoms can diffuse a sufficient distance at a high temperature, thus the atoms can diffuse to the favorable lattice positions eventually. 

Furthermore, the bulk diffusion also exists in the crystal and the waveguide layer would generate the process of recrystallization, which would reduce the density of the defects and improve the c-axis preferred orientation. When the sputtering temperature at 200 °C, the probability of atomic desorption is very high because the adsorption life of the external particles is short. The result leads to part of the waveguide layer surface being rich zinc and a large number of oxygen vacancies existing in the waveguide layer. Finally, the internal defects of the waveguide layer would increase, and the c-axis orientation become poor, while the stress would also increase accordingly. The defects are fewest and internal stress is smallest of the ZnO waveguide layer fabricated at 150 °C confirmed by the slope of the line at 150 °C is smaller than that of the other lines. Therefore, the film quality is better relatively.

### 4.3. The Response Characteristics of the Love Wave Device

**Figure 7:**
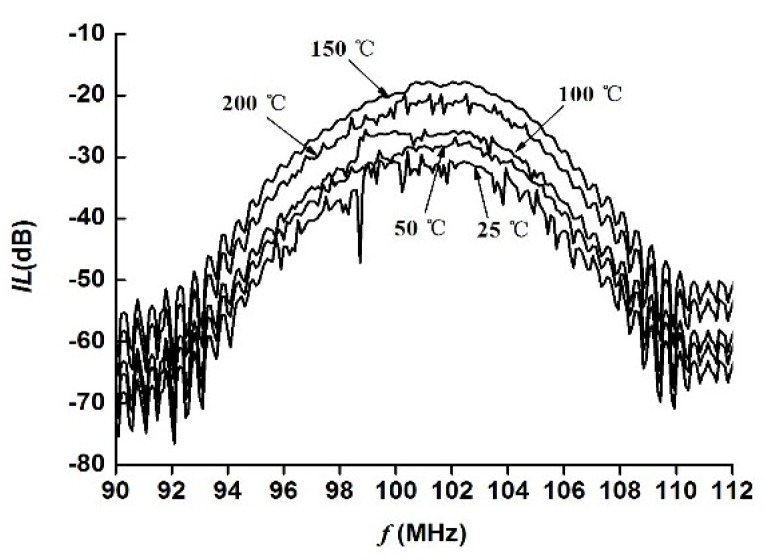
Response characteristics of the Love wave device.

The response characteristics of the Love wave device with ZnO waveguide layer fabricated at different sputtering temperatures are measured by network analyzer. The result is shown in Figure 7. The horizontal and longitudinal axes in Figure 7 are the frequency and insertion loss of the Love wave device, respectively.

The Figure 7 reveals that the Love wave device fabricated at a lower sputtering temperature has a large fluctuation near the center frequency. With the sputtering temperature increasing, the fluctuation in the band is decreasing but it again increases at 200 °C. Thus, the Love device fabricated at 150 °C has a better ripple suppression effect in the band.

**Figure 8:**
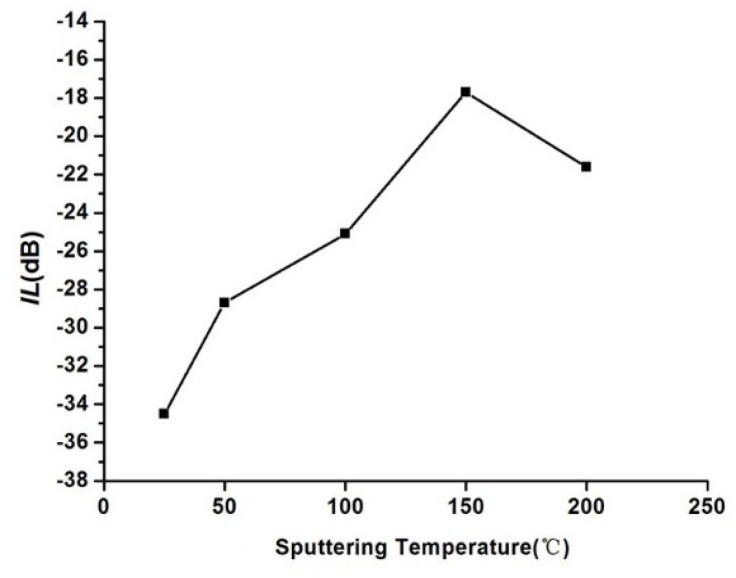
Relationship between the sputtering temperature and the insertion loss.

The insertion loss of the Love wave device is one of the indexes that reflect the performance of the device. Figure 8 shows the effect of the sputtering temperature on the insertion loss.

It can be seen from Figure 8 that the Love wave device has the lowest insertion loss of −17.7 dB at 150 °C, and the insertion loss of the devices is less than −20 dB at the other sputtering temperatures. The results caused by the absorption effect of the ZnO waveguide layer material to the acoustic wave is enhancing. According to the analysis of the ZnO waveguide layer above, the ZnO waveguide layer fabricated at 150 °C has good c-axis preferential orientation, few internal defects and small stress. The delay would exist between the surface and the bottom part of the ZnO waveguide layer when the acoustic wave propagating, which leads to the strain of the longitudinal direction (thickness direction) in the waveguide layer. If the ZnO waveguide itself has a relatively high strain, the insertion loss of the device would increase. Therefore, the device has the lowest insertion loss at the sputtering temperature is 150 °C.

### 4.4. Characteristic Analysis of the Humidity Sensor

The center frequency of the Love wave humidity sensor is 102.561 MHz under the normal temperature and humidity. After adjusting the humidity of the closed air chamber to a relative value, the difference between the center frequency of the sensor under this humidity and that under the normal temperature and humidity is regarded as the output signal of the sensor.

The specific measurement process of humidity as follows.

(1) The semiconductor temperature regulator is started to control the temperature of the air chamber at 25 °C, and the temperature of the closed air chamber should be kept at 25 °C throughout the experiment.
(2) The relative humidity of the closed air chamber is reduced to 10% by the silica gel desiccant and then keeps for 300 s. The system completes the measurement of the relative humidity when the output signal of the system reaches a stable value.
(3) Each relative humidity was measured by the same method.

**Figure 9:**
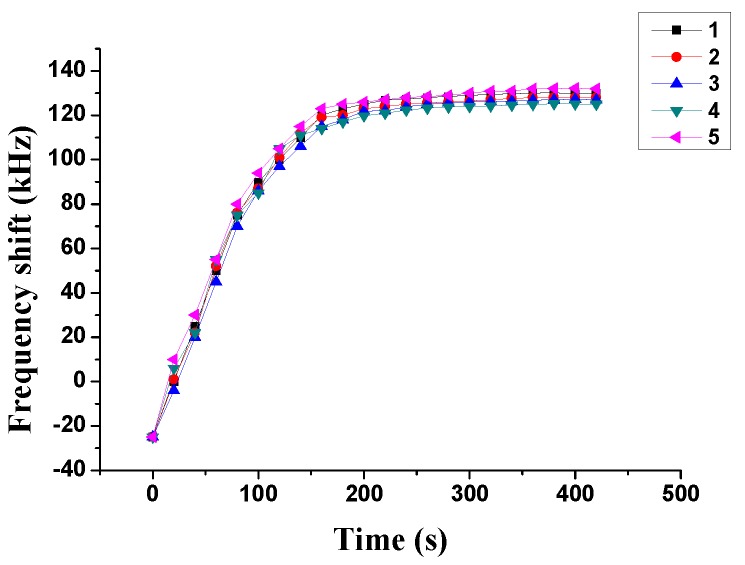
Repeatability of the Love wave humidity sensor at 60 %RH.

Repeatability is an important characteristic of the sensor. To study the repeatability of the Love wave humidity sensor with the ZnO waveguide layer, the relative humidity of 60% was measured three times by the Love wave humidity sensor. The test result is shown in Figure 9. In Figure 9, the positive frequency shift represents the increase of the center frequency, and the negative frequency shift represents the decrease of the center frequency. Since the frequency shift is defined as the difference between the center frequency of the sensor under a certain humidity and the center frequency under normal humidity at same temperature. The decrease of the center frequency is due to the humidity in the closed air chamber is lower than normal humidity and the influence of humidity on the ZnO waveguide layer is weakened. On the contrary, when the humidity is higher than the normal humidity, the central frequency of the device will increase.

From Figure 9, it can be concluded that the frequency shift of the sensor is changing as the time pass. The trends of the five curves show that the response of the sensor to relative humidity is slowly changing. With the time increasing, the frequency shift of the sensor is increase gradually. Then it tends to be steady at 200 s and reaches a steady value at 300 s. According to the experimental results, the trends of the three curves are same basically, especially when the time is 0–90 s and 180–420 s. The results illustrate that the sensor with ZnO waveguide layer has a good repeatability. If the fabrication process of the ZnO waveguide layer is improved under the same film thickness, the repeatability of the sensor can be further improved.

**Figure 10:**
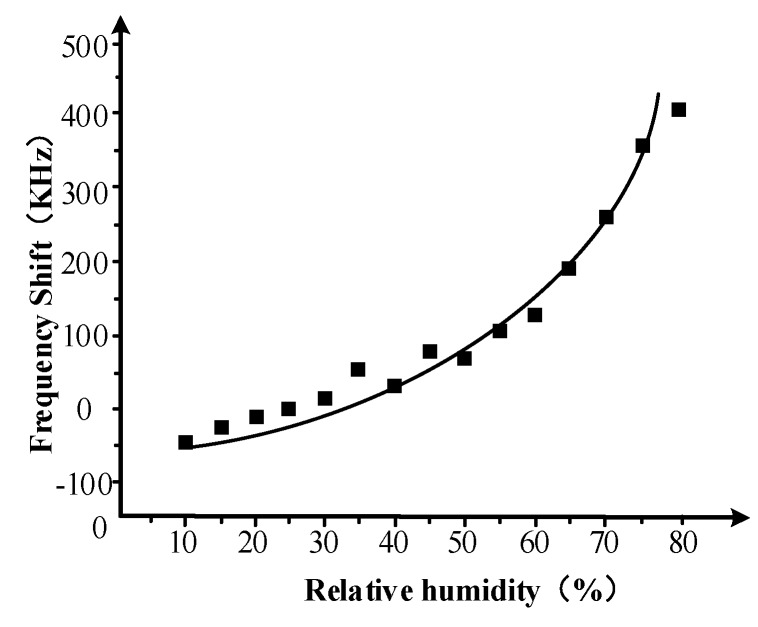
Frequency shift of the sensor change with the relative humidity.

To study the relationship between the relative humidity and the frequency shift of the sensor, the humidity of the closed air chamber is increased from 10% to 80% stepped with the relative humidity interval of 5%. The frequency shift of the sensor is measured five times at each relative humidity, and then the average value is considered to be the frequency shift at that humidity. The steady value of the system output is recorded and saved at each relative humidity. The result is shown in Figure 10.

The result of Figure 10 shows that the frequency of the sensor has a one-to-one relationship with the relative humidity of the measuring chamber. In general, the frequency shift of the Love wave humidity sensor is increasing with the relative humidity increasing. The frequency shift is small and has a linear relationship nearly with the relative humidity when the relative humidity is less than 30%. The change in the frequency shift becomes quickly when the relative humidity increases from 40% to 60%. After the relative humidity goes over 60%, the change of the frequency shift becomes increasingly quick, so the slight relative humidity change would lead to very large frequency shift.

The sensitivity of the sensor is about 6.43 kHz/RH when the relative humidity is in the range of 10% to 80%. In addition, the frequency resolution of the network analyzer is 1 Hz, so the Love wave humidity sensor can detect a change of 0.00015% of the relative humidity theoretically. That is, the test limitation of the sensor is 0.00015 RH/Hz. Therefore, the sensor has high sensitivity and good application prospect.

**Figure 11:**
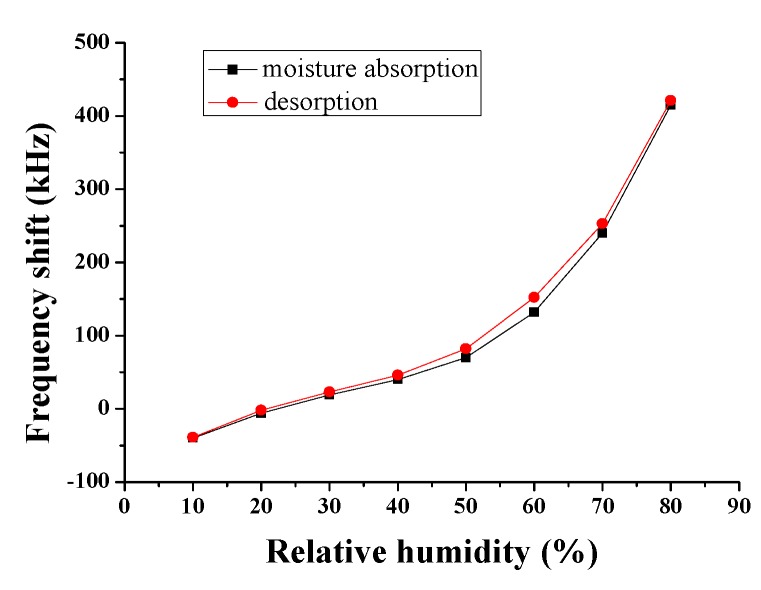
Hysteresis characteristics of the Love wave humidity sensor.

The moisture absorption and desorption of the Love wave humidity sensor need time; thus, the frequency shift of the sensor will be different in the process of the moisture absorption and desorption when the relative humidity is different, and the other conditions are the same. The hysteresis characteristics were measured at 25 °C and the results are shown in Figure 11.

According to Figure 11, the trends of the frequency shift in the process of desorption and moisture absorption is almost the same. The change in the frequency shift is largest and the largest hysteresis error is 6 %RH when the relative humidity is 60%. The result shows that the hysteresis of the sensor is good.

**Figure 12:**
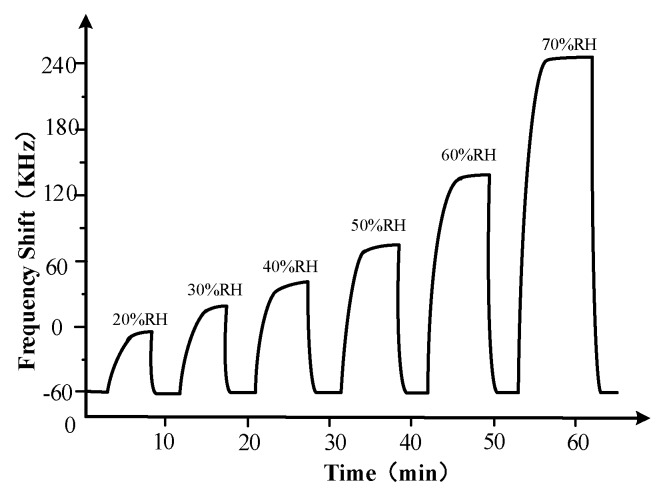
Humidity response characteristics of the Love wave humidity sensor.

The humidity response characteristics of the Love wave humidity sensor were measured at 25 °C and the result is shown in Figure 12. It can be seen from Figure 12 that the frequency shifts of the sensor were −6 kHz, 19 kHz, 40 kHz, 73 kHz, 135 kHz and 244 kHz when the relative humidity increased from 10% to 20%, 30%, 40%, 50%, 60% and 70%, respectively. The 80% response time is less than 3 min and all the recovery time is less than 30 s, which indicates that the Love wave humidity sensor has good response characteristics.

**Figure 13:**
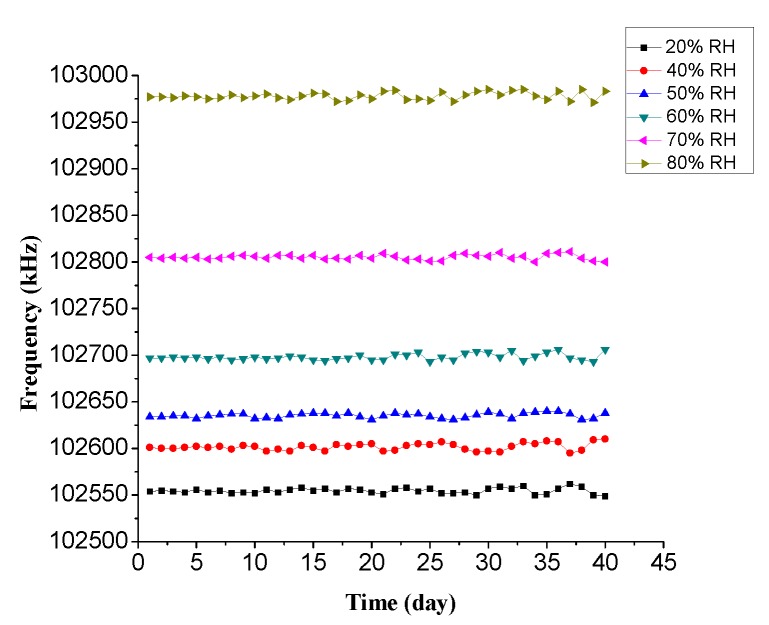
Stability of the Love wave humidity sensor.

To investigate the long-term stability of the Love wave humidity sensor, the sensor was placed in circumstances with relative humidity at 20%, 40%, 50%, 60%, 70% and 80% for 40 days, respectively. The result is shown in Figure 13. As show in Figure 13, the frequency fluctuation of the sensor is less than 5% in 40 days under different relative humidity, which indicated that the sensor had good long-term stability.

## 5. Conclusions

In this paper, a fabrication scheme of the ZnO waveguide layer in the Love wave device by the magnetron sputtering technique is presented. The influence of the different sputtering temperatures in the performance of ZnO waveguide layer and the Love wave device is discussed. Then the crystal orientation, grain size and stress of the ZnO waveguide layer were measured and analyzed. Furthermore, a humidity measurement system is designed and the characteristics of the Love wave device with ZnO waveguide layer as humidity sensor are analyzed. The results show that the performance of the Love wave device is improved firstly and then is degraded when the sputtering temperature increases from 25 °C to 200 °C. Meanwhile, the c-axis orientation is highest, and the internal defects are least of the ZnO waveguide layer fabricated at 150 °C. In addition, the Love wave humidity sensor with ZnO waveguide layer fabricated at 150 °C has good reproducibility, fine long-term stability and high sensitivity.

## Figures and Tables

**Table 1:** Design parameters of the Love device.

IDTi	Electrode width/μm	4.888
	Electrode gap/μm	4.888
	Number of electrode pairs	128
	Maximum acoustic aperture/mm	1.5212
Multistrip coupler (MSC)	Electrode width/μm	7.16
	Electrode gap/μm	7.16
	Number of electrode pairs	103
IDTo	Electrode width/μm	4.888
	Electrode gap/μm	4.888
	Number of electrode pairs	128
	Maximum acoustic aperture /mm	1.5212

**Table 2:** Sputtering parameters.

Sputtering Parameter	Value
Power	120 W
Time	200 min
Pressure	1.2 Pa
Temperature	150 °C

**Table 3:** Microstructure parameters of the ZnO waveguide layers at the different sputtering temperatures.

*T_s_*/°C	*η*/rad	*D*/nm
25	0.00401	37.76
50	0.00370	40.93
100	0.00334	45.35
150	0.00284	53.36
200	0.00313	48.41
